# Functional Targets for Epstein-Barr Virus BART MicroRNAs in B Cell Lymphomas

**DOI:** 10.3390/cancers16203537

**Published:** 2024-10-19

**Authors:** Devin N. Fachko, Bonnie Goff, Yan Chen, Rebecca L. Skalsky

**Affiliations:** Vaccine and Gene Therapy Institute, Oregon Health and Science University, Beaverton, OR 97006, USA

**Keywords:** microRNAs, Epstein-Barr virus, B cell lymphoma, BART miRNAs

## Abstract

MicroRNAs are key post-transcriptional regulators of gene expression and their dysregulation is often linked to cancer. Epstein-Barr virus encodes 22 BamHI A Rightward Transcript (BART) miRNAs, which are expressed in nearly all EBV-associated cancers and implicated in viral pathogenesis. To investigate biological targets for BART miRNAs in B cell lymphomas, we performed a meta-analysis of publicly available Ago-CLIP datasets from EBV-positive Burkitt lymphomas (BLs), primary effusion lymphomas (PELs), AIDS-associated diffuse large B cell lymphomas (DLBCLs), and lymphoblastoid cell lines (LCLs). Our analysis focused on comparing targets of EBV BART miRNAs across the different types of transformed B cells. Using reporter assays, we then experimentally validated over 50 functional interactions between BART miRNAs and cellular protein-coding transcripts involved in activities such as B cell differentiation (*PRDM1*, *IRF4*, and *MYC*), cell cycle regulation (*UHMK1, CDKN1A, MDM2*, and *NPA*T), apoptosis (*MCL1*), signaling and intracellular trafficking (*GAB1, SOS1, MAPK1, RAB11A, CAV1*, and *RANBP9*), and tumor suppression (*CCDC6*). Moreover, ectopic BART miRNA expression in several EBV-negative BL cells induced transcriptional changes that may influence molecular signatures of EBV-associated BLs. Collectively, our findings reveal novel, functional interactions for BART miRNAs in lymphomas and provide insights into their roles in these B cell cancers.

## Simple Summary

Epstein-Barr virus (EBV) latently infects over 90% of adults worldwide and is linked to several cancers such as Burkitt lymphoma. Amongst the few factors expressed by the EBV during latent infection are microRNAs (miRNAs) that can regulate both viral and cellular gene expression. In this study, we examined cellular targets of EBV miRNAs. Through bioinformatics and molecular analysis, we provide evidence of 52 new functional interactions for EBV miRNAs with protein-coding transcripts involved in B cell differentiation, cell cycle, and intracellular trafficking. Collectively, these data provide a resource for EBV miRNA targets and yield important insight into functional roles for these viral factors in B cell cancers.

## 1. Introduction

Epstein-Barr virus (EBV) infection is associated with several B cell lymphoproliferative disorders and hematological malignancies, including Hodgkin lymphoma (HL), non-Hodgkin lymphoma (NHL), Burkitt lymphoma (BL), and diffuse large B cell lymphoma (DLBCL) [1,2]. Initially isolated from pediatric BL biopsies, EBV is a human g-herpesvirus that infects over 90% of adults worldwide. EBV infection is usually asymptomatic or causes mononucleosis that resolves in a few weeks; however, in some individuals, malignancies that are linked to the virus occur [1]. BL is the most common B cell lymphoma in children and is an aggressive B cell neoplasm that is often genetically characterized by chromosomal translocations that lead to deregulated Myc expression [3,4,5]. Three epidemiological BL variants have been described: endemic BL (eBL), sporadic BL (sBL), and immunodeficiency-associated BL (iBL). Unlike sBL, for which only 10–20% are EBV-positive [6], eBL is predominantly observed in malaria holoendemic regions and linked to infection with EBV in over 90% of cases [4]. Lymphomas such as BL, HL, and DLBCL in immunodeficient individuals are associated with poorer prognosis when infected with EBV [7].

EBV was the first herpesvirus found to encode viral microRNAs (miRNAs) [8], which, akin to their cellular counterparts, act as potent post-transcriptional regulators of gene expression. The EBV genome harbors 25 precursor miRNAs encoded within two major regions. Three BHRF1 pre-miRNAs flank the BHRF1 protein-coding gene, while two large clusters of miRNAs are in the BART region. Initial studies indicated that a subset of BART miRNAs may be elevated in BL and HL biopsies compared to normal latently infected cells [9]. Further profiling studies on tumor tissues revealed that BART miRNAs account for a large proportion of the total miRNA population within EBV-positive lymphomas [10,11,12,13]. Analysis of clinical biopsies showed high levels of BART miRNAs in AIDS-related lymphomas, whereas HL had lower levels [14]. EBV BART miRNAs were also found to be abundant in BL samples; in 62 out of 69 BL cases, EBV miRNAs comprised >20% of all detected miRNAs [15].

Two TATA-less promoters, P1 and P2, have been described for the BART miRNAs [16]. Early studies reported conflicting results as to whether all BART miRNAs are upregulated during lytic induction. In initial experiments, Akata cells treated with anti-IgG and B95–8 cells treated with TPA exhibited little to no correlation between BART miRNA levels and replicating EBV genomes [17], whereas treatment of MutuI, Akata, and other EBV+ BL cell lines with 5-aza-Cd led to overall increases in BART miRNA expression [18]. Consistent with the latter, our prior miRNA sequencing studies revealed that all BART miRNAs are highly upregulated in EBV-infected BL cells when induced to reactivate with antibodies that cross-link the B cell receptor (BCR) [19].

Whole EBV genome sequencing following hybrid capture of viruses isolated from patients with EBV-associated diseases (CAEBV, ENKTL, DLBCL) has revealed frequent deletions in the BART miRNA regions [20,21,22]. A 313 bp deletion corresponding to the miR-BART12 encoding region was recently identified in a classic Hodgkin’s lymphoma (cHL) [23]. In contrast to B cell and T/NK cell lymphomas, BART defective viruses are rarely observed in epithelial cancers [24,25]. Intact EBV genomes are typically present in individuals with primary infectious mononucleosis or post-transplant lymphoproliferative disease (PTLD), indicating that a complete BART region is needed for these conditions [25,26,27].

Exact functions for EBV BART miRNAs are continuing to emerge; however, numerous studies point to roles in immune regulation, protection of infected cells from apoptosis, and activities in lytic reactivation [11,28,29,30,31]. In previous work, we demonstrated that a handful of BART miRNAs (miR-BART2, BART17, and BART18) attenuated signaling through the BCR [32], presumably putting the brakes on lytic reactivation. However, once BCR-mediated signals have triggered EBV reactivation, at least one miRNA, miR-BART9, supported the lytic cycle by dampening FOXO3 expression [19]. Deletion of the BART miRNAs from EBV strain M81 led to increased BZLF1 expression and enhanced tumor growth in immunocompromised mice [33]. In humanized mouse models, BART miRNA knockout viruses displayed heightened lytic gene expression [34]. These phenotypes may be attributed in part to the multiple EBV transcripts that are regulated by BART miRNAs, such as BZLF1/BRLF1, BHRF1, and BALF5 [35,36,37]. Notably, EBV B95–8, a common laboratory strain, lacks most BART miRNAs. Reintroducing the 17 missing BART miRNAs into EBV B95–8 recombinants augmented lymphoblastoid cell line (LCL) outgrowth [38], whereas expression of BART miRNAs in BL cell lines complemented the loss of EBV genomes and reduced apoptosis [30]. This anti-apoptotic effect is thought to be mediated through the suppression of cellular targets like *CASP3*, Bim, and *BBC3/PUMA* [30,39,40]. In eBL samples, inverse correlations between BART miRNA levels and potential target RNA transcripts have been observed [11,41]; however, direct biochemical evidence for the regulation of these differentially expressed genes remains lacking.

Despite the examples above, few studies have been directed toward experimentally validating cellular targets for BART miRNAs in B cell lymphomas and determining their mechanistic roles in the development of these malignancies. Accurate identification of viral miRNA targets is an ongoing challenge and requires systematic approaches. Our recent assessment of EBV BART miRNA interactions in DLBCLs using CLIP-based approaches revealed multiple targets related to type I interferon signaling pathways [28]. While hundreds of additional BART miRNA interactions were identified in DLBCLs, these have not been fully investigated beyond the initial study. In this study, we sought to investigate BART miRNA targets first by interrogating published Argonaute-crosslinking and immunoprecipitation (Ago-CLIP) datasets of transformed B cells and, subsequently, using functional assays to confirm molecular interactions. Our results reveal novel cellular targets relevant to B cell lymphomagenesis and, importantly, provide experimental evidence of these interactions. We also assessed transcriptional changes in BART miRNA-expressing BL cells and detected decreases in multiple BART miRNA targets that potentially influence molecular signatures of EBV-associated BL.

## 2. Materials and Methods

### 2.1. Cell Culture and Transfections

HEK293T cells were maintained at 37 °C in Dulbecco’s modified Eagle’s medium supplemented with 10% fetal bovine serum (FBS), penicillin, and streptomycin. LCLs and BL cell lines (BJAB, Ramos, BL2, and BL41-B95.8) were maintained at 37 °C in RPMI supplemented with 15% FBS, penicillin, and streptomycin. BL41-B95.8 cells are persistently infected with EBV B95.8 and originated from the laboratory of Dr. Elliott Kieff. EBV-negative BL-derived BL2 cells were provided by Dr. Robert White and originated from the laboratory of Dr. Martin Allday. BJAB and Ramos are EBV-negative BL-derived cell lines [42,43]. LCLD2 and LCLWT are previously described [44]. HEK293T cells were transfected using Lipofectamine 2000 (ThermoFisher Scientific, Waltham, MA, USA) according to the manufacturer’s protocol. For the preparation of lentiviruses, HEK293T cells were plated in 15 cm plates in complete media and transfected using polyethylenimine (PEI) with 15 µg of lentivector, 9 µg of pDeltaR8.75, and 6 µg of pMD2G. Medium was changed to complete RPMI 1640 at between 16 and 24 hr post-transfection. Lentiviral particles were harvested by sterile filtration of the supernatant using a 0.22 µm filter at 48 hr and 96 hr post-transfection and used to transduce 1 **×** 10^6 BL cells.

### 2.2. RNA Isolation and qRT-PCR

Total RNA was isolated from cells using TRIzol (ThermoFisher Scientific, Waltham, MA, USA) according to the manufacturer’s protocol, except substituting the wash step with 95% ethanol. To measure gene expression, RNA was DNase-treated and reverse transcribed using MultiScribe (ThermoFisher Scientific, Waltham, MA, USA) with random hexamers. Cellular transcripts were detected using qPCR primers to gene-specific regions and PowerUp SYBR green qPCR mastermix (Thermo Fisher Scientific, Waltham, MA, USA). Oligonucleotide sequences are available upon request. The miRNAs were measured with TaqMan qRT-PCR assays, using miR−16 as an internal control. All qPCR reactions were performed in technical replicates, and relative quantities were calculated using the 2−ΔΔCt method.

### 2.3. Luciferase Assays

Cloning of 3′UTRs and dual luciferase reporter assays were performed as previously described [19]. Briefly, HEK293T cells were plated in 96-well black-well plates and co-transfected with 20 ng of 3′ UTR reporter and 250 ng of control vector (pLCE) or miRNA expression vector using Lipofectamine 2000 (Thermo Fisher Scientific, Waltham, MA, USA). Cells were harvested in 1x passive lysis buffer (Promega) at 48–72 hr post-transfection, and lysates were assayed for luciferase activity using the dual luciferase reporter assay system (Promega) and a luminometer. All values are reported as relative light units (RLU) relative to luciferase internal control and normalized to the pLCE control vector.

### 2.4. Bioinformatics

Bioinformatics analysis of Ago-CLIP datasets (Table S1) was performed using the Galaxy platform [45] (https://usegalaxy.org/ accessed on 25 August 2024). Sequencing datasets were obtained from the NCBI sequence read archive (SRA), uploaded to Galaxy, and assessed for read quality using FastQC. Reads were trimmed of Illumina sequencing adapters using TrimGalore. Following adapter removal, reads were mapped to the HG19 human genome using Bowtie [46] for Illumina (-*n* 3 for PC or 2 for HC, -l 17, -m 25, --best, --strata). Subsequent BAM files were imported into PIPE-CLIP v1.0.0 [47], and Ago interaction sites were defined using default parameters. BEDOPS [48] tools were used to define overlapping CLIP sites between datasets. Analysis of PAR-CLIP (photo-activatable ribonucleoside cross-linking and immunoprecipitation) datasets using PARalyzer was performed as previously described [28,29,49], requiring at least three reads per cluster. Seed matches (≥7mer1A) to EBV miRNAs within Ago-CLIP sites were annotated *ad hoc.*

### 2.5. PAR-CLIP

Established LCLs (LCLD2 and LCLWT) [44] were expanded in the log phase and cultured in 100 uM 4-SU (4-thiouridine) for 24 hr prior to cross-linking at UV 365 nm. Ago-bound RNAs were immunopurified using antibodies to Ago2 (Abcam) as previously described [28,35,50]. The cDNA libraries were sequenced on the Illumina platform (accessed by 25 August 2024), and raw sequencing reads were obtained using FASTQ format.

### 2.6. Statistical Analysis

Statistical analysis was performed using Microsoft Excel and GraphPad Prism v10. Data are presented as average ± standard deviation (SD). For luciferase and qRT-PCR assays, differences between groups were analyzed using Student’s *t*-tests to determine statistical significance, and *p*-values less than 0.05 were considered significant.

## 3. Results

### 3.1. Ago Interactome Across Multiple Types of Transformed B Cells

MiRNA: mRNA relationships can be inferred from gene expression analysis and/or sequence predictions; however, such approaches are limited in their capabilities to demonstrate interactions between a miRNA and target transcript. As Ago-CLIP-based techniques provide direct experimental evidence of Ago-mediated interactions with target RNAs, we therefore chose to investigate miRNA targets within EBV-infected B cells by performing meta-analysis of 41 Ago-CLIP datasets representing different transformed B cell types: EBV+ LCLs, EBV+ BL, EBV/KSHV+ PEL, and EBV+ AIDS-associated DLBCL cell lines (Table S1) [28,35,50,51,52,53,54,55]. The controls included in this analysis were EBV-negative BL and EBV-neg/KSHV+ PEL. Ago CLIP reads were aligned to the human genome using Bowtie [46] for Illumina in Galaxy [45], and subsequently, high-resolution Ago-interaction sites were defined.

**Figure 1:**
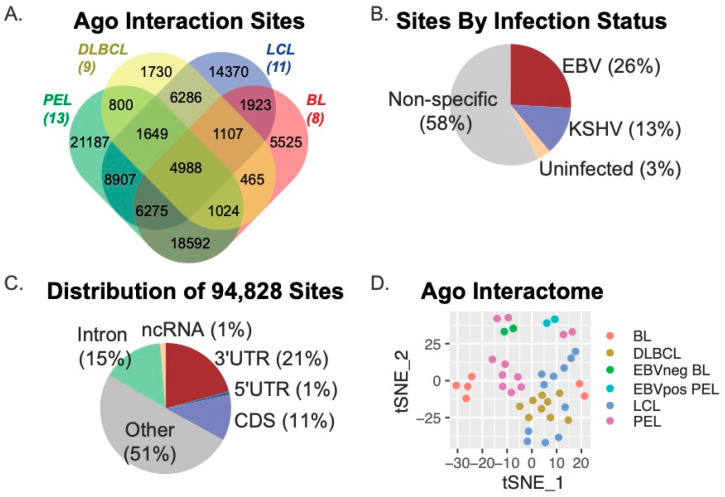
**Meta-analysis of Ago-CLIP interactions in EBV-transformed B cells.** (**A**) Distribution of Ago interaction sites across various transformed B cell lines. Ago interaction sites were identified in 41 Ago-CLIP libraries using PIPE-CLIP. A total of 94,828 unique sites were found, of which 52,043 were found in at least two cell types, and 4988 were found in all cell types. (**B**) Distribution of Ago interaction sites by viral infection status. A total of 26% of sites were specific to B cells infected with EBV only, and 13% were specific to B cells infected with KSHV. (**C**) Distribution of unique Ago interaction sites mapping to human genes. Annotation of Ago-CLIP interaction sites determined that 21% mapped to the 3′UTRs of protein-coding genes, 11% mapped to the CDS, (coding sequence) and 51% mapped to non-protein coding regions of the human genome. (**D**) The tSNE method was applied to the 16,020 genes harboring Ago interaction sites to visualize the similarities and differences of the Ago interactions across the various B cell types.

We initially determined Ago interaction sites using PIPE-CLIP v1.1.0, an open-source tool that accommodates analysis of multiple CLIP-based methods [47]. Thus, both Ago-HITS-CLIP (i.e., Jijoye BL, BCBL−1 PEL) and Ago-PAR-CLIP (i.e., DLBCL, LCL) datasets could be compared through this analysis platform to derive common and unique Ago-accessible sites. Amongst the 41 Ago-CLIP samples, we identified a total of 602,764 Ago-interaction sites, which were parsed further with BEDOPS tools [48] to define 94,828 unique sites present in two or more samples (Figure 1). Figure 1A shows the distribution of the 94,828 Ago interaction sites according to B cell type (DLBCL, LCL, PEL, or BL). A total of 4988 sites were common to all cell types, while 52,043 sites (54.9% of the total sites) were present in two or more cell types. We evaluated viral infection status and found that 26% of sites were specific to B cells harboring only EBV infection, while 13% were specific to B cells infected with Kaposi’s sarcoma-associated herpesvirus (KSHV) (Figure 1B.) Annotation of the 94,828 Ago-interaction sites revealed that 21% (19,109 sites) mapped to the 3′UTRs of protein-coding genes, 11% mapped to coding regions, and 51% mapped to non-protein coding regions of the human genome (other) (Figure 1C). Of the 4988 Ago-interaction sites detected in all cell types, 787 were present in 3′UTRs. In total, the 94,828 Ago-interaction sites were mapped to 16,020 different human genes.

To visualize similarities and differences within the Ago interactomes between B cell types, we applied the t-distributed Stochastic Neighbor Embedding (t-SNE) method to the 16,020 human genes that were captured in the 41 Ago-CLIP datasets. Consistent with current models that describe LCLs as phenotypically similar to AIDS-associated DLBCLs [2,56] as well as post-transplant lymphoproliferative disorders (PTLD) that can occur in immunocompromised transplant patients [57], the DLBCL and LCL Ago interactomes were found to be the most similar (Figure 1D).

### 3.2. EBV miRNAs Target > 4000 Protein-Coding Transcripts in Transformed B Cells

**Figure 2:**
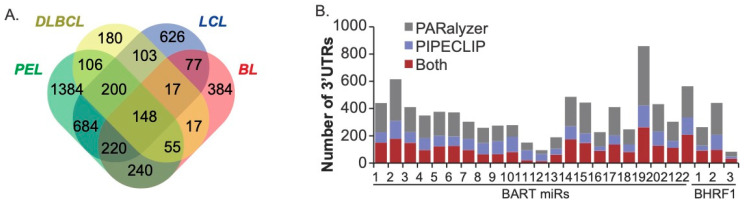
**EBV miRNA targets in transformed B cells.** (**A**) Distribution of EBV miRNA targets in the EBV-positive Ago-CLIP datasets. 3′UTRs were scanned for canonical seed matches to the BART and BHRF1 miRNAs. A total of 1869 targets were found in two or more cell types, and 148 targets were found in all cell types. (**B**) The number of 3′UTR targets assigned to each EBV miRNA using both PARalyzer and PIPECLIP. An average of 355 cellular genes were detected for each pre-miRNA.

To evaluate EBV miRNA interactions, the EBV-positive Ago-CLIP datasets (IBL1, IBL4, BCKN1, all LCLs, Jijoye, BC1) were examined further. PARalyzer was used to query additional high-resolution Ago-interaction sites within the PAR-CLIP datasets [49]. Focusing on the Ago interactions, specifically within protein-coding transcripts that were defined by both PIPE-CLIP and PARalyzer, we scanned 3′UTR sites for canonical seed matches (≥7mer1A) to all the EBV BART and BHRF1 miRNAs. On a gene level, 4443 total EBV miRNA targets were defined, with 1869 targets detected in two or more cell types (Figure 2). Notably, we identified 148 protein-coding transcripts that were commonly targeted by EBV miRNAs in all four B cell types (Figure 2A, Table S2) and thus represent the highest confidence targets. Amongst these are previously validated EBV miRNA targets such as *CAND1, CASP3, CDKN1A, CLIC4, DAZAP2, DICER1, GAB1, IPO7, PAK2, PRDM1, PTEN, SIAH1*, and *TOMM22*.

Assessment of the total number of targets assigned to each EBV miRNA revealed an average of 355 cellular genes per pre-miRNA. Since each pre-miRNA potentially gives rise to two mature miRNAs, this averages to ~177 targets per viral miRNA, which is in range with estimates for cellular miRNAs [58]. Overall, 2771 protein-coding transcripts were identified by both bioinformatics tools that harbored binding sites for EBV miRNAs. Interestingly, BART11, BART12, BART13, and BHRF1–3 had fewer cellular targets than the other EBV miRNAs (Figure 2B). The low level of targets for miR-BHRF1–3 is not entirely surprising, given that previous studies revealed unusual binding characteristics for this miRNA [35]. Moreover, this miRNA is not expressed in PEL or BL [17,51], has a low abundance in LCLs compared to other EBV miRNAs, and is known to regulate EBV BZLF1 [59]. Although further work is needed, the possible regulation of EBV rather than cellular transcripts may also explain the fewer number of cellular targets for the three BART miRNAs.

To investigate cellular pathways regulated by EBV miRNAs in transformed B cells, we used the open-source, manually curated pathway database Reactome [60] to analyze the full list of EBV miRNA targets. The top 18 pathways significantly enriched (*p* < 0.05, FDR < 0.05) for EBV miRNA targets are listed in Table 1. Of particular interest for tumorigenesis are targets involved in Cell Cycle (R-HSA−1640170), Cell Cycle Checkpoints (R-HSA−69620), and Cellular Senescence (R-HSA−2559583).

### 3.3. Validation of EBV BART miRNA Interactions by Luciferase Reporter Assays

To determine whether the compiled list of EBV BART miRNA targets indeed has functional activity, we sought to experimentally validate interactions by cloning 3′UTRs of select genes of interest into luciferase reporter vectors. Reporters were then co-transfected into HEK293T cells with individual EBV BART miRNA expression vectors, and luciferase expression was measured. In prior work, we have focused our efforts on characterizing EBV miRNA targets related to NF-kappaB signaling [50], type I IFN signaling [28], or BCR signaling [32]. Here, we expanded our efforts to investigate target interactions that (i) were previously shown to be regulated by the EBV miRNAs expressed from EBV B95–8 but include sites for other BART miRNAs or (ii) have not previously been interrogated and are of interest to virus-mediated lymphomagenesis.

**Figure 3:**
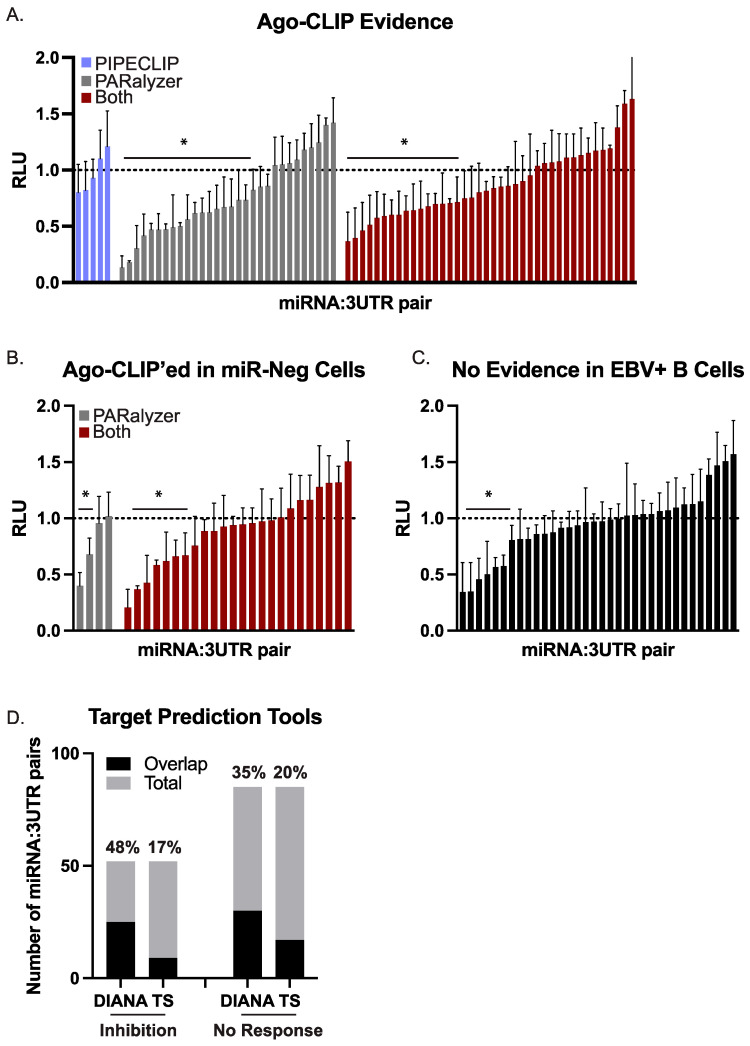
**Validation of BART miRNA targets by luciferase reporter assays.** HEK293T cells were co-transfected with a 3′UTR luciferase reporter and either a BART miRNA expression vector or an empty control vector. After 48–72 h post-transfection, cells were lysed and assayed for dual luciferase activity. For each 3′UTR reporter, luciferase values in the presence of a given miRNA are shown relative to the empty control vector (pLCE). The reported average is at least three independent experiments. RLU = relative light units. By Student’s *t*-test, * *p* < 0.05. (**A**) 75 miRNA:3UTR pairs for which there is biochemical evidence of an interaction by Ago-CLIP. (**B**) 28 miRNA:3UTR pairs for which there is biochemical evidence of interaction by Ago-CLIP; however, the site was also detected in miRNA-negative cells. (**C**) 34 miRNA:3UTR pairs that were tested as controls. (**D**) EBV miRNA targets were predicted by either DIANA microT or TargetScan. Shown is the overlap of predicted targets with the number of miRNA:3UTR pairs exhibiting luciferase inhibition or showing no response.

We tested 103 different miRNA:3UTR interactions that were identified in Ago-CLIP datasets for 20 different BART miRNAs and 43 different 3′UTRs. No targets for BART15 or BART20 were tested. Amongst the miRNA:3UTR interactions chosen for assessment were 11 (*CDK6, CDKN1A, CLIC4, GAB1, MAPK1, MCL1, MDM2, MYC, PRDM1, PTEN, UHMK1*) of the 148 high confidence targets that were present in all B cell types (Figure 2A and Figure 3A). Within this list, we included five genes that were recurrently within the top 18 enriched cellular pathways (Table 1) and related to cell cycle and cellular senescence (*CDKN1A, CDK6, MYC, MDM2, MAPK1*).

miRNA:3UTR pairs were divided into two groups. In the first group, we tested 75 miRNA:3UTR pairs for which there was evidence of interaction only in B cells that expressed a given EBV miRNA. For example, EBV B95–8 LCLs lack 16 of the BART miRNAs. In several instances, we detected an Ago interaction site in EBV B95–8 LCLs that overlapped with an Ago interaction site in wild-type EBV infected B cells and could be assigned to one of the 16 BART miRNAs missing from EBV B95–8. While normally we would flag these interactions as false positives, we instead opted to test several of these miRNA:3UTR pairs in a second group to determine whether these might be function sites.

Of the 75 combinations tested in the first group, 36 combinations (48%) yielded significant luciferase inhibition (Figure 3A), confirming these as EBV miRNA targets. We then evaluated the 28 miRNA:3UTR pairs for which there was Ago-CLIP evidence of interactions in B cells that are not expected to express the viral miRNA (Figure 3B). Of the 28 miRNA:3UTR pairs tested, nine (32%) resulted in significant knockdown in luciferase reporter assays (Figure 3B). As controls, we further tested 34 miRNA:3UTR pairs for which no biochemical evidence of interaction by Ago-CLIP was found (Figure 3C). The 3′UTRs included in this control set represent potential targets of an EBV BART miRNA but were tested against BART miRNAs that were not expected to confer knockdown. Within this set, seven of the 34 miRNA:3UTR pairs (21%) showed significant knockdown.

To further investigate the seven miRNA:3UTR pairs in the control group that conferred luciferase inhibition, we used two computational tools (TargetScan Custom v5.2, DIANA microT v2023) [61,62] and predicted EBV BART miRNA targets. Two of the seven miRNA:3UTR pairs (BART7:SOS1, BART22:SIAH1) were captured in DIANA microT predictions, providing an explanation for the observed knockdown. To more broadly evaluate all 137 different miRNA:3UTR combinations, we compared the results of the luciferase assays to the lists of EBV miRNA targets predicted by either DIANA microT or TargetScan. The number of miRNA:3UTR combinations overlapping with each list of predicted targets is shown in Figure 3D. By Fisher’s exact test (*p* = 0.154 for DIANA microT, *p* = 0.823 for TargetScan), no significant enrichments were identified in the group of 52 miRNA:3UTR pairs showing knockdown compared to the group of 85 miRNA:3UTR pairs for which there was no response. We also compared our list of functionally validated EBV BART miRNA:mRNA pairs to CLASH-identified miRNA:mRNA hybrids present in Akata BL cells [15]. Surprisingly, very little overlap was found with the 137 sites tested—in fact, only one interaction (*RAB11A* with miR-BART18–5p) yielding significant knockdown was captured by CLASH. Collectively, these findings demonstrate the continuing need for in vitro, rather than in silico, biochemical methods to examine viral miRNA target interactions.

Taken together, our assessment of 137 different combinations of BART miRNAs and human 3′UTRs identified 52 combinations that yielded > 20% knockdown, demonstrating these as functional EBV miRNA target sites. Direct biochemical evidence by Ago-CLIP was documented for 45 of these sites, while two additional 3′UTR targets could be explained by a predicted canonical seed match (≥7mer1A) to either EBV miR-BART7 or miR-BART22.

### 3.4. Targets of EBV BART miRNAs Are Involved in B Cell Differentiation, Cell Cycle, and Intracellular Trafficking

**Figure 4:**
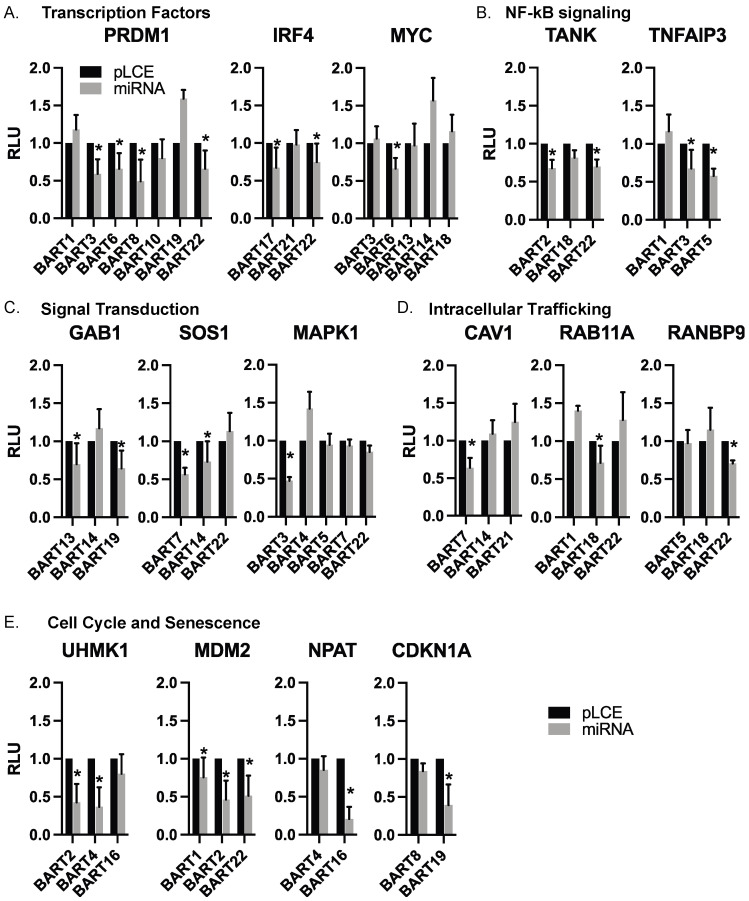
**Individual EBV BART miRNA target interactions.** 3′UTR interactions are shown with individual EBV BART miRNAs. Luciferase assays are from Figure 3, with targets arranged according to biological function. Values are reported relative to the pLCE empty control vector. RLU = relative light units. By Student’s *t*-test, * *p* < 0.05.

Through our luciferase reporter assays, we validated cellular targets of EBV BART miRNAs that are known to be involved in B cell differentiation (*PRDM1, IRF4, MYC),* regulation of the cell cycle (*UHMK1, CDKN1A, MDM2, NPAT*), signal transduction (*GAB1, SOS1, MAPK1*), NF-kB signaling (*TANK, TNFAIP3*), apoptosis (*MCL1*), and intracellular trafficking (*RAB11A, CAV1, RANBP9*). Extending findings from our previous analysis of EBV miRNA targets related to IFN signaling [28], we also validated *IRF9, IFIT2,* and *IFNAR2*. The complete list of genes evaluated in luciferase assays is reported in Table S3. Table S4 lists the glossary of genes evaluated in this study. Figure 4 highlights categories of targets with similar biological functions and shows the individual 3′UTR reporters tested against various EBV BART miRNAs.

### 3.5. EBV BART miRNAs Suppress Target Transcripts in BL Cells

**Figure 5:**
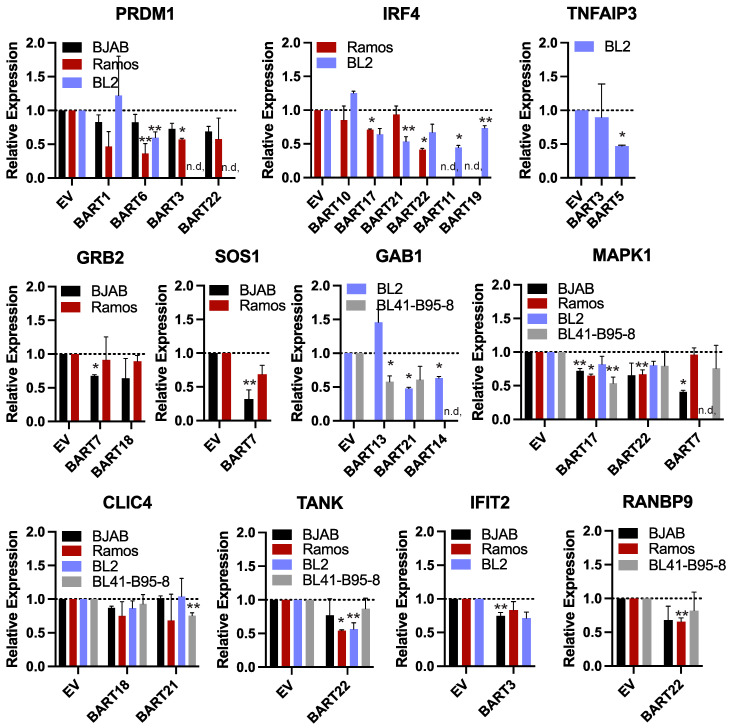
**Reduced gene expression levels in the presence of EBV BART miRNAs.** RNA was harvested from BL cell lines stably expressing individual EBV BART miRNAs or empty vector (EV). Transcript levels of indicated EBV miRNA targets were assessed by qRT-PCR. Values are normalized to GAPDH and reported relative to control cells (EV) for each BL cell line. *n*.d. = not determined. By Student’s *t*-test, ** *p* < 0.1, * *p* < 0.05.

Inverse correlations between BART miRNA levels and potential target RNA transcripts have been reported in BL [11,41]; however, biochemical evidence of these interactions as direct miRNA targets remains to be demonstrated. We examined the differentially expressed, downregulated genes that were identified in EBV-positive BL compared to EBV-negative BL from previous studies [11,41] and found only three genes that overlapped with EBV BART miRNA targets from Figure 2. Thus, to investigate cellular gene expression that potentially correlates with viral miRNA levels, we generated 28 different EBV-negative BL cell lines that stably express a total of 13 individual EBV BART miRNAs. BJAB, Ramos, and BL2 cell lines were transduced with pLCE-based lentiviral vectors that express the different BART miRNAs [50], and green fluorescent protein (GFP) expression was used to monitor transduction efficiency. To further evaluate the effects of EBV, we also engineered BL41 cells infected with EBV B95–8 to express multiple BART miRNAs that are not encoded in EBV B95–8. Total RNA was harvested from BL cells 2–3 weeks post-transduction, and levels of indicated genes were measured by qRT-PCR (Figure 5).

We focused our analysis on 11 genes that were found to respond to various EBV BART miRNAs in luciferase assays. Select genes included the following: (i) *PRDM1* and *IRF4*, which are master transcriptional regulators of B cell differentiation and play key roles in the EBV latent/lytic switch [63]; (ii) *TNFAIP3* which encodes A20 (an inhibitor of NF-kB activation) and is mutated in multiple cancers including DLBCLs [64]; (iii) *GRB2, GAB1*, and *SOS1* which are involved in receptor tyrosine kinase signal transduction as well as *MAPK1* which encodes Erk2 involved in MAPK signaling [65,66]; (iv) *CLIC4* which encodes the multifunctional chloride intracellular channel 4 protein that also has roles in inflammation [67]; (v) *TANK* which encodes a bifunctional adaptor protein that either activates, or inhibits NF-kB [68]; (vi) *IFIT2* which is expressed in response to IFN signaling; (vii) *RANBP9* which activates Ras signaling by recruiting SOS proteins and can also bind EBV Zta and enhance Zta-dependent transcriptional activity [69].

While expression of each of these genes was variable amongst the different BL cell lines, we observed modest down-regulation of all 11 genes tested in response to an EBV BART miRNA (** *p* < 0.1), and seven of these genes were significantly reduced (* *p* < 0.05, Figure 5). Of the 52 miRNA:3UTR pairs that showed inhibition in luciferase assays, we found 12 miRNA:3UTR combinations that yielded significant reductions at the RNA level. Notably, a significant knockdown of IRF4 was observed in response to BART17 and BART22, which both suppressed the *IRF4* luciferase reporter (Figure 4 and Figure 5). Similarly, *PRDM1* levels were reduced by BART3 and BART6, which both suppressed the *PRDM1* reporter (Figure 4 and Figure 5).

## 4. Discussion

EBV infection is implicated in a wide range of hematological malignancies and lymphoproliferative disorders. While many studies have focused on roles for BART miRNAs in EBV-positive nasopharyngeal and gastric carcinomas, roles for BART miRNAs in EBV-positive B cell cancers are incompletely understood. In this study, we sought to increase the number of confirmed EBV BART miRNA targets by functionally evaluating interactions that were identified through Ago-CLIP methods in EBV+ B cell lymphomas. miRNA:3′UTR target pairs were selected based upon relevance to B cell tumorigenesis and investigated using luciferase reporter assays as well as gene expression. Through our analysis, we found that in transformed B cells, EBV BART miRNAs regulate multiple B cell transcription factors (i.e., *IRF4, PRDM1, MYC*), signal transducers that act downstream of growth factor receptors (i.e., *GAB1, SOS1, MAPK1, TANK, TNFAIP3*), and several cell cycle regulators (*UHMK1, CDKN1A, MDM2, NPAT*).

Defining EBV miRNA targets remains an ongoing challenge. Previous microarray analysis of BL cells expressing multiple BART miRNAs identified only seven candidate targets [30], and recent efforts to evaluate protein changes in response to individual BART miRNAs (i.e., miR-BART7 and miR-BART9) reported only a few—specifically, 17 targets for BART7 and nine targets for BART9 [70]. Despite conservation with other lymphocryptovirus miRNAs [50], EBV miRNAs predominantly lack sequence similarities to human miRNAs. The absence of conservation features limits the use of most target prediction algorithms [61,62,71]. Indeed, of the 52 EBV miRNA interactions that conferred knockdown in reporter assays, only 25 (48%) of these were predicted by DIANA-microT, whereas only nine (17%) were predicted by TargetScan (Figure 3).

Further complicating target analysis is the fact that a handful of EBV miRNAs do exhibit seed sequence homology to cellular miRNAs. For example, we have previously shown that miR-BART9–3p can regulate cellular targets that overlap with the miR−141/200 family [19]. However, despite their common seed sequences, these miRNAs also regulate their own independent targets, which was only revealed through functional assays [19]. In this study, we evaluated multiple targets of miR-BART22, which has sequence homology to primate-specific miR−524–5p and miR−520d−5p. Both miR−524–5p and miR−520d−5p are members of the chromosome 19 miRNA cluster (C19MC) that are involved in cellular reprogramming and potentially involved in tumorigenesis through enhancement of cell proliferation and suppression of apoptosis [72]. At present, the extent to which miR-BART22-targeted transcripts overlap with miR−524–5p targets remains unclear; however, given the unique roles of C19MC miRNAs, future studies are warranted to determine if miR-BART22 plays a similar role in reprogramming.

Two key targets of EBV BART miRNAs with strong relevance to transcriptional reprogramming and B cell fates are *PRDM1* (Blimp1) and *IRF4*. These encode transcription factors that play crucial roles in the survival and differentiation of long-term plasma cells [73,74]. IRF4 exhibits biphasic expression during early B cell development and again in mature germinal center (GC) B cells following B cell receptor engagement [75]. *PRDM1* is transcriptionally regulated by IRF4 and often mutated in DLBCL [76]. Notably, a subset of EBV-associated DLBCLs and BLs share characteristics of GC cells originating from either the dark or light zone [1,11,77]. Within the dark zone of the GC, antigen-activated B cells proliferate rapidly and undergo somatic hypermutation [78]. In the light zone, interactions with follicular helper T cells and dendritic cells facilitate selection and affinity maturation of GC B cells, enabling differentiation into memory B cells or long-lived antibody-producing plasma cells [78]. With regards to EBV infection, Blimp1 can induce lytic gene expression by activating the EBV Zta promoter [79]. Alterations in IRF4 levels can also impact EBV latency [80]. Within LCLs, loss of IRF4 expression promotes the EBV lytic cycle, whereas within BL cell lines, the EBV lytic cycle can be induced by increasing the levels of IRF4 [80,81], indicating that cellular context and differentiation state contribute significantly to these phenotypes. As EBV-infected B cells must navigate complex B cell transcriptional programs within the GC, the regulation of *IRF4* and *PRDM1* by multiple BART miRNAs likely plays a key role in this process.

One outstanding question that remains is whether sequence variations in EBV BART miRNAs impact their abilities to functionally regulate cellular targets. Given the varying prevalence of EBV-associated malignancies worldwide, it has been hypothesized that naturally occurring sequence variations in EBV strains may contribute to pathogenesis [20,21,25,26,82]. With respect to the BARTs, three major sequence subtypes have been identified for the BART miRNA cluster 2 region [25], while at least six major subtypes have been documented for the BART miRNA cluster 1 region [82]. These BART miRNA cluster subtypes exhibit differences in geographical distributions and are further differentially associated with risk for NPC and other EBV-associated malignancies [25,82]. While many natural sequence variations are documented in the primary BART transcripts, most nucleotide changes occur within regions outside of the precursor miRNA sequences [25,82]. A few exceptions include (i) EBV miR-BART19–5p which exhibits a T to C nucleotide polymorphism at nt 17 in C666 and strains from other NPC cell lines compared to EBV strains derived from B cells [83], (ii) the BART6 precursor which exhibits deletions and expansions within a T-rich region of the terminal loop (TL) [84], (iii) the BART7 precursor which exhibits a U to A change in the TL [25,26], and (iv) the BART17 precursor which exhibits a C to U change in the TL [25,26]. Importantly, all these single nucleotide variations (SNVs) are outside of the miRNA seed regions and, therefore, are not predicted to have major impacts on the specificity of target transcripts.

Nucleotide differences within precursor miRNAs can potentially impact mature miRNA expression levels; however, further studies show that SNVs outside of BART pre-miRNA sequences alter expression levels [25,82]. Nucleotide polymorphisms in regions flanking the BART22 precursor miRNA impact its processing by Drosha [85]. Notably, SNVs within a region between BART11 and BART12 impact the expression of these miRNAs [25]. As our analysis revealed that BART11 and BART12 have the least number of cellular targets in the BL, PEL, and DLBCL lines analyzed (Figure 2), one explanation may lie in EBV sequence differences within this region. Further sequence analysis of these and additional EBV strains will be needed to fully investigate the functionality of SNVs within the BART region.

## 5. Conclusions

In the present study, we demonstrate 52 functional EBV BART miRNA interactions that occur within 32 different 3′UTRs of cellular protein-coding transcripts, representing one of the largest datasets of functionally validated EBV miRNA targets. Our targeted gene expression data support the idea that EBV interferes with host transcriptional programs through viral miRNAs and highlights B cell transcription factors such as PRDM1 and IRF4 as noteworthy components that necessitate further investigation. Together, these data provide an important resource for future studies evaluating how EBV BART miRNAs contribute to B cell reprogramming during EBV infection and determining how these interactions influence B cell lymphomagenesis.

## Figures and Tables

**Table 1:** REACTOME pathways enriched for EBV miRNA targets.

Identifier	Pathway	Entities	*p*-Value	FDR
R-HSA−74160	Gene expression (Transcription)	54	2.17E−08	8.27E−06
R-HSA−73857	RNA Polymerase II Transcription	52	3.17E−09	1.81E−06
R-HSA−212436	Generic Transcription Pathway	50	1.50E−09	1.72E−06
R-HSA−1280215	Cytokine Signaling	33	4.39E−06	6.11E−04
R-HSA−8953897	Cellular responses to stimuli	32	4.81E−06	6.11E−04
R-HSA−2262752	Cellular responses to stress	32	3.37E−06	5.50E−04
R-HSA−1640170	Cell Cycle	22	2.09E−04	0.00707
R-HSA−449147	Signaling by Interleukins	20	3.38E−04	0.00947
R-HSA−3700989	Transcriptional Regulation by TP53	23	9.10E−08	2.59E−05
R-HSA−9006931	Signaling by Nuclear Receptors	14	5.35E−04	0.01298
R-HSA−9006925	Intracellular signals	15	1.04E−04	0.00491
R-HSA−1257604	PIP3 activates AKT signaling	14	9.01E−05	0.00441
R-HSA−5688426	Deubiquitination	12	3.97E−04	0.01073
R-HSA−69620	Cell Cycle Checkpoints	11	0.00105	0.01988
R-HSA−8939211	ESR-mediated signaling	12	1.34E−04	0.00581
R-HSA−2559583	Cellular Senescence	12	1.22E−05	0.00139
R-HSA−6807070	PTEN Regulation	10	7.63E−05	0.00389

## Data Availability

PAR-CLIP datasets for LCLD2 and LCLWT are available through NCBI Sequence Read Archive under BioProject PRJNA1171868.

## Supplementary Materials

The following supporting information can be downloaded at: https://www.mdpi.com/article/10.3390/cancers16203537/s1, Table S1: Ago-CLIP datasets analyzed in this study; Table S2: 148 high confidence targets for EBV miRNAs with references for confirmed targets; Table S3: 137 miRNA:3UTR pairs tested in luciferase assays; Table S4: Glossary of genes.

## References

[1] Farrell P.J. (2019). Epstein-Barr Virus and Cancer. Annu. Rev. Pathol..

[2] Carbone A., Cesarman E., Spina M., Gloghini A., Schulz T.F. (2009). HIV-associated lymphomas and gamma-herpesviruses. Blood.

[3] Dalla-Favera R., Bregni M., Erikson J., Patterson D., Gallo R.C., Croce C.M. (1982). Human c-myc onc gene is located on the region of chromosome 8 that is translocated in Burkitt lymphoma cells. Proc. Natl. Acad. Sci. USA.

[4] Moormann A.M., Bailey J.A. (2016). Malaria—How this parasitic infection aids and abets EBV-associated Burkitt lymphomagenesis. Curr. Opin. Virol..

[5] Zech L., Haglund U., Nilsson K., Klein G. (1976). Characteristic chromosomal abnormalities in biopsies and lymphoid-cell lines from patients with Burkitt and non-Burkitt lymphomas. Int. J. Cancer.

[6] Mbulaiteye S.M., Biggar R.J., Bhatia K., Linet M.S., Devesa S.S. (2009). Sporadic childhood Burkitt lymphoma incidence in the United States during 1992–2005. Pediatr Blood Cancer.

[7] Stuhlmann-Laeisz C., Borchert A., Quintanilla-Martinez L., Hoeller S., Tzankov A., Oschlies I., Kreuz M., Trappe R., Klapper W. (2016). In Europe expression of EBNA2 is associated with poor survival in EBV-positive diffuse large B-cell lymphoma of the elderly. Leuk. Lymphoma.

[8] Pfeffer S., Zavolan M., Grasser F.A., Chien M., Russo J.J., Ju J., John B., Enright A.J., Marks D., Sander C. (2004). Identification of virus-encoded microRNAs. Science.

[9] Qiu J., Cosmopoulos K., Pegtel M., Hopmans E., Murray P., Middeldorp J., Shapiro M., Thorley-Lawson D.A. (2011). A novel persistence associated EBV miRNA expression profile is disrupted in neoplasia. PLoS Pathog..

[10] Motsch N., Alles J., Imig J., Zhu J., Barth S., Reineke T., Tinguely M., Cogliatti S., Dueck A., Meister G. (2012). MicroRNA profiling of Epstein-Barr virus-associated NK/T-cell lymphomas by deep sequencing. PLoS ONE.

[11] Piccaluga P.P., Navari M., De Falco G., Ambrosio M.R., Lazzi S., Fuligni F., Bellan C., Rossi M., Sapienza M.R., Laginestra M.A. (2016). Virus-encoded microRNA contributes to the molecular profile of EBV-positive Burkitt lymphomas. Oncotarget.

[12] Imig J., Motsch N., Zhu J.Y., Barth S., Okoniewski M., Reineke T., Tinguely M., Faggioni A., Trivedi P., Meister G. (2011). microRNA profiling in Epstein-Barr virus-associated B-cell lymphoma. Nucleic Acids Res..

[13] Oduor C.I., Movassagh M., Kaymaz Y., Chelimo K., Otieno J., Ong’echa J.M., Moormann A.M., Bailey J.A. (2017). Human and Epstein-Barr Virus miRNA Profiling as Predictive Biomarkers for Endemic Burkitt Lymphoma. Front. Microbiol..

[14] Sakamoto K., Sekizuka T., Uehara T., Hishima T., Mine S., Fukumoto H., Sato Y., Hasegawa H., Kuroda M., Katano H. (2017). Next-generation sequencing of miRNAs in clinical samples of Epstein-Barr virus-associated B-cell lymphomas. Cancer Med..

[15] Ungerleider N., Bullard W., Kara M., Wang X., Roberts C., Renne R., Tibbetts S., Flemington E.K. (2021). EBV miRNAs are potent effectors of tumor cell transcriptome remodeling in promoting immune escape. PLoS Pathog..

[16] Chen H., Huang J., Wu F.Y., Liao G., Hutt-Fletcher L., Hayward S.D. (2005). Regulation of expression of the Epstein-Barr virus BamHI-A rightward transcripts. J. Virol..

[17] Pratt Z.L., Kuzembayeva M., Sengupta S., Sugden B. (2009). The microRNAs of Epstein-Barr Virus are expressed at dramatically differing levels among cell lines. Virology.

[18] Kim D.N., Song Y.J., Lee S.K. (2011). The role of promoter methylation in Epstein-Barr virus (EBV) microRNA expression in EBV-infected B cell lines. Exp. Mol. Med..

[19] Chen Y., Fachko D.N., Ivanov N.S., Skalsky R.L. (2021). B Cell Receptor-Responsive miR-141 Enhances Epstein-Barr Virus Lytic Cycle via FOXO3 Inhibition. mSphere.

[20] Okuno Y., Murata T., Sato Y., Muramatsu H., Ito Y., Watanabe T., Okuno T., Murakami N., Yoshida K., Sawada A. (2019). Defective Epstein-Barr virus in chronic active infection and haematological malignancy. Nat. Microbiol..

[21] Mabuchi S., Hijioka F., Watanabe T., Yanagi Y., Okuno Y., Masud H., Sato Y., Murata T., Kimura H. (2021). Role of Epstein-Barr Virus C Promoter Deletion in Diffuse Large B Cell Lymphoma. Cancers.

[22] Peng R.J., Han B.W., Cai Q.Q., Zuo X.Y., Xia T., Chen J.R., Feng L.N., Lim J.Q., Chen S.W., Zeng M.S. (2019). Genomic and transcriptomic landscapes of Epstein-Barr virus in extranodal natural killer T-cell lymphoma. Leukemia.

[23] Kawatsuki A., Igawa T., Urata T., Tanaka T., Sato Y., Yoshino T. (2020). Deletion of BART miRNA-encoding cluster in Epstein-Barr virus DNA in classic Hodgkin lymphoma. Pathol. Int..

[24] Kimura H., Okuno Y., Sato Y., Watanabe T., Murata T. (2021). Deletion of Viral microRNAs in the Oncogenesis of Epstein-Barr Virus-Associated Lymphoma. Front. Microbiol..

[25] Correia S., Palser A., Elgueta Karstegl C., Middeldorp J.M., Ramayanti O., Cohen J.I., Hildesheim A., Fellner M.D., Wiels J., White R.E. (2017). Natural Variation of Epstein-Barr Virus Genes, Proteins, and Primary MicroRNA. J. Virol..

[26] Palser A.L., Grayson N.E., White R.E., Corton C., Correia S., Ba Abdullah M.M., Watson S.J., Cotten M., Arrand J.R., Murray P.G. (2015). Genome diversity of Epstein-Barr virus from multiple tumor types and normal infection. J. Virol..

[27] Yajima M., Kakuta R., Saito Y., Kitaya S., Toyoda A., Ikuta K., Yasuda J., Ohta N., Kanda T. (2021). A global phylogenetic analysis of Japanese tonsil-derived Epstein-Barr virus strains using viral whole-genome cloning and long-read sequencing. J. Gen. Virol..

[28] Bouvet M., Voigt S., Tagawa T., Albanese M., Chen Y.A., Chen Y., Fachko D.N., Pich D., Gobel C., Skalsky R.L. (2021). Multiple Viral microRNAs Regulate Interferon Release and Signaling Early during Infection with Epstein-Barr Virus. mBio.

[29] Chen Y., Kincaid R.P., Bastin K., Fachko D.N., Skalsky R.L. (2024). MicroRNA-focused CRISPR/Cas9 screen identifies miR-142 as a key regulator of Epstein-Barr virus reactivation. PLoS Pathog..

[30] Vereide D.T., Seto E., Chiu Y.F., Hayes M., Tagawa T., Grundhoff A., Hammerschmidt W., Sugden B. (2014). Epstein-Barr virus maintains lymphomas via its miRNAs. Oncogene.

[31] Albanese M., Tagawa T., Buschle A., Hammerschmidt W. (2017). MicroRNAs of Epstein-Barr Virus Control Innate and Adaptive Antiviral Immunity. J. Virol..

[32] Chen Y., Fachko D., Ivanov N.S., Skinner C.M., Skalsky R.L. (2019). Epstein-Barr virus microRNAs regulate B cell receptor signal transduction and lytic reactivation. PLoS Pathog..

[33] Lin X., Tsai M.H., Shumilov A., Poirey R., Bannert H., Middeldorp J.M., Feederle R., Delecluse H.J. (2015). The Epstein-Barr Virus BART miRNA Cluster of the M81 Strain Modulates Multiple Functions in Primary B Cells. PLoS Pathog..

[34] Deng Y., Munz C. (2021). Roles of Lytic Viral Replication and Co-Infections in the Oncogenesis and Immune Control of the Epstein-Barr Virus. Cancers.

[35] Skalsky R.L., Corcoran D.L., Gottwein E., Frank C.L., Kang D., Hafner M., Nusbaum J.D., Feederle R., Delecluse H.J., Luftig M.A. (2012). The viral and cellular microRNA targetome in lymphoblastoid cell lines. PLoS Pathog..

[36] Barth S., Pfuhl T., Mamiani A., Ehses C., Roemer K., Kremmer E., Jaker C., Hock J., Meister G., Grasser F.A. (2008). Epstein-Barr virus-encoded microRNA miR-BART2 down-regulates the viral DNA polymerase BALF5. Nucleic Acids Res..

[37] Jung Y.J., Choi H., Kim H., Lee S.K. (2014). MicroRNA miR-BART20-5p stabilizes Epstein-Barr virus latency by directly targeting BZLF1 and BRLF1. J. Virol..

[38] Seto E., Moosmann A., Gromminger S., Walz N., Grundhoff A., Hammerschmidt W. (2010). Micro RNAs of Epstein-Barr virus promote cell cycle progression and prevent apoptosis of primary human B cells. PLoS Pathog..

[39] Marquitz A.R., Mathur A., Nam C.S., Raab-Traub N. (2011). The Epstein-Barr Virus BART microRNAs target the pro-apoptotic protein Bim. Virology.

[40] Kang D., Skalsky R.L., Cullen B.R. (2015). EBV BART MicroRNAs Target Multiple Pro-apoptotic Cellular Genes to Promote Epithelial Cell Survival. PLoS Pathog..

[41] Oduor C.I., Kaymaz Y., Chelimo K., Otieno J.A., Ong’echa J.M., Moormann A.M., Bailey J.A. (2017). Integrative microRNA and mRNA deep-sequencing expression profiling in endemic Burkitt lymphoma. BMC Cancer.

[42] Menezes J., Leibold W., Klein G., Clements G. (1975). Establishment and characterization of an Epstein-Barr virus (EBC)-negative lymphoblastoid B cell line (BJA-B) from an exceptional, EBV-genome-negative African Burkitt’s lymphoma. Biomedicine.

[43] Klein G., Giovanella B., Westman A., Stehlin J.S., Mumford D. (1975). An EBV-genome-negative cell line established from an American Burkitt lymphoma; receptor characteristics. EBV infectibility and permanent conversion into EBV-positive sublines by in vitro infection. Intervirology.

[44] Skinner C.M., Ivanov N.S., Barr S.A., Chen Y., Skalsky R.L. (2017). An Epstein-Barr Virus MicroRNA Blocks Interleukin-1 (IL-1) Signaling by Targeting IL-1 Receptor 1. J. Virol..

[45] Galaxy C. (2024). The Galaxy platform for accessible, reproducible, and collaborative data analyses: 2024 update. Nucleic Acids Res..

[46] Langmead B., Trapnell C., Pop M., Salzberg S.L. (2009). Ultrafast and memory-efficient alignment of short DNA sequences to the human genome. Genome Biol..

[47] Chen B., Yun J., Kim M.S., Mendell J.T., Xie Y. (2014). PIPE-CLIP: A comprehensive online tool for CLIP-seq data analysis. Genome Biol..

[48] Neph S., Kuehn M.S., Reynolds A.P., Haugen E., Thurman R.E., Johnson A.K., Rynes E., Maurano M.T., Vierstra J., Thomas S. (2012). BEDOPS: High-performance genomic feature operations. Bioinformatics.

[49] Corcoran D.L., Georgiev S., Mukherjee N., Gottwein E., Skalsky R.L., Keene J.D., Ohler U. (2011). PARalyzer: Definition of RNA binding sites from PAR-CLIP short-read sequence data. Genome Biol..

[50] Skalsky R.L., Kang D., Linnstaedt S.D., Cullen B.R. (2014). Evolutionary conservation of primate lymphocryptovirus microRNA targets. J. Virol..

[51] Gottwein E., Corcoran D.L., Mukherjee N., Skalsky R.L., Hafner M., Nusbaum J.D., Shamulailatpam P., Love C.L., Dave S.S., Tuschl T. (2011). Viral microRNA targetome of KSHV-infected primary effusion lymphoma cell lines. Cell Host Microbe.

[52] Majoros W.H., Lekprasert P., Mukherjee N., Skalsky R.L., Corcoran D.L., Cullen B.R., Ohler U. (2013). MicroRNA target site identification by integrating sequence and binding information. Nat. Methods.

[53] Erhard F., Haas J., Lieber D., Malterer G., Jaskiewicz L., Zavolan M., Dolken L., Zimmer R. (2014). Widespread context dependency of microRNA-mediated regulation. Genome Res..

[54] Riley K.J., Rabinowitz G.S., Yario T.A., Luna J.M., Darnell R.B., Steitz J.A. (2012). EBV and human microRNAs co-target oncogenic and apoptotic viral and human genes during latency. EMBO J..

[55] Haecker I., Gay L.A., Yang Y., Hu J., Morse A.M., McIntyre L.M., Renne R. (2012). Ago HITS-CLIP expands understanding of Kaposi’s sarcoma-associated herpesvirus miRNA function in primary effusion lymphomas. PLoS Pathog..

[56] Nichele I., Zamo A., Bertolaso A., Bifari F., Tinelli M., Franchini M., Stradoni R., Aprili F., Pizzolo G., Krampera M. (2012). VR09 cell line: An EBV-positive lymphoblastoid cell line with in vivo characteristics of diffuse large B cell lymphoma of activated B-cell type. PLoS ONE.

[57] Martinez O.M., Krams S.M. (2017). The Immune Response to Epstein Barr Virus and Implications for Posttransplant Lymphoproliferative Disorder. Transplantation.

[58] Friedman R.C., Farh K.K., Burge C.B., Bartel D.P. (2009). Most mammalian mRNAs are conserved targets of microRNAs. Genome Res..

[59] Fachko D.N., Chen Y., Skalsky R.L. (2022). Epstein-Barr Virus miR-BHRF1-3 Targets the BZLF1 3′UTR and Regulates the Lytic Cycle. J. Virol..

[60] Milacic M., Beavers D., Conley P., Gong C., Gillespie M., Griss J., Haw R., Jassal B., Matthews L., May B. (2024). The Reactome Pathway Knowledgebase 2024. Nucleic Acids Res..

[61] Tastsoglou S., Alexiou A., Karagkouni D., Skoufos G., Zacharopoulou E., Hatzigeorgiou A.G. (2023). DIANA-microT 2023: Including predicted targets of virally encoded miRNAs. Nucleic Acids Res..

[62] Lewis B.P., Burge C.B., Bartel D.P. (2005). Conserved seed pairing, often flanked by adenosines, indicates that thousands of human genes are microRNA targets. Cell.

[63] Kenney S.C., Mertz J.E. (2014). Regulation of the latent-lytic switch in Epstein-Barr virus. Semin. Cancer Biol..

[64] Consortium A.P.G. (2017). AACR Project GENIE: Powering Precision Medicine through an International Consortium. Cancer Discov..

[65] Li N., Batzer A., Daly R., Yajnik V., Skolnik E., Chardin P., Bar-Sagi D., Margolis B., Schlessinger J. (1993). Guanine-nucleotide-releasing factor hSos1 binds to Grb2 and links receptor tyrosine kinases to Ras signalling. Nature.

[66] Perez-Baena M.J., Cordero-Perez F.J., Perez-Losada J., Holgado-Madruga M. (2023). The Role of GAB1 in Cancer. Cancers.

[67] Domingo-Fernandez R., Coll R.C., Kearney J., Breit S., O’Neill L.A.J. (2017). The intracellular chloride channel proteins CLIC1 and CLIC4 induce IL-1beta transcription and activate the NLRP3 inflammasome. J. Biol. Chem..

[68] Pomerantz J.L., Baltimore D. (1999). NF-kappaB activation by a signaling complex containing TRAF2, TANK and TBK1, a novel IKK-related kinase. EMBO J..

[69] Yang Y.C., Feng T.H., Chen T.Y., Huang H.H., Hung C.C., Liu S.T., Chang L.K. (2015). RanBPM regulates Zta-mediated transcriptional activity in Epstein-Barr virus. J. Gen. Virol..

[70] Caetano B.F.R., Rocha V.L., Rossini B.C., Dos Santos L.D., Elgui De Oliveira D. (2024). Epstein-Barr Virus miR-BARTs 7 and 9 modulate viral cycle, cell proliferation, and proteomic profiles in Burkitt lymphoma. Tumour Virus Res..

[71] Agarwal V., Bell G.W., Nam J.W., Bartel D.P. (2015). Predicting effective microRNA target sites in mammalian mRNAs. eLife.

[72] Nguyen P.N.N., Choo K.B., Huang C.J., Sugii S., Cheong S.K., Kamarul T. (2017). miR-524-5p of the primate-specific C19MC miRNA cluster targets TP53IPN1- and EMT-associated genes to regulate cellular reprogramming. Stem Cell Res. Ther..

[73] Minnich M., Tagoh H., Bonelt P., Axelsson E., Fischer M., Cebolla B., Tarakhovsky A., Nutt S.L., Jaritz M., Busslinger M. (2016). Multifunctional role of the transcription factor Blimp-1 in coordinating plasma cell differentiation. Nat. Immunol..

[74] Tellier J., Shi W., Minnich M., Liao Y., Crawford S., Smyth G.K., Kallies A., Busslinger M., Nutt S.L. (2016). Blimp-1 controls plasma cell function through the regulation of immunoglobulin secretion and the unfolded protein response. Nat. Immunol..

[75] Ochiai K., Maienschein-Cline M., Simonetti G., Chen J., Rosenthal R., Brink R., Chong A.S., Klein U., Dinner A.R., Singh H. (2013). Transcriptional regulation of germinal center B and plasma cell fates by dynamical control of IRF4. Immunity.

[76] Pasqualucci L., Compagno M., Houldsworth J., Monti S., Grunn A., Nandula S.V., Aster J.C., Murty V.V., Shipp M.A., Dalla-Favera R. (2006). Inactivation of the PRDM1/BLIMP1 gene in diffuse large B cell lymphoma. J. Exp. Med..

[77] Frontzek F., Staiger A.M., Wullenkord R., Grau M., Zapukhlyak M., Kurz K.S., Horn H., Erdmann T., Fend F., Richter J. (2023). Molecular profiling of EBV associated diffuse large B-cell lymphoma. Leukemia.

[78] Mesin L., Ersching J., Victora G.D. (2016). Germinal Center B Cell Dynamics. Immunity.

[79] Reusch J.A., Nawandar D.M., Wright K.L., Kenney S.C., Mertz J.E. (2015). Cellular differentiation regulator BLIMP1 induces Epstein-Barr virus lytic reactivation in epithelial and B cells by activating transcription from both the R and Z promoters. J. Virol..

[80] Bristol J.A., Brand J., Ohashi M., Eichelberg M.R., Casco A., Nelson S.E., Hayes M., Romero-Masters J.C., Baiu D.C., Gumperz J.E. (2022). Reduced IRF4 expression promotes lytic phenotype in Type 2 EBV-infected B cells. PLoS Pathog..

[81] Gao Y., Wang L., Lei Z., Li J., Forrest J.C., Liang X. (2019). IRF4 promotes Epstein-Barr virus activation in Burkitt’s lymphoma cells. J. Gen. Virol..

[82] Wang Q., He H., Ji X., Liu Y., Li H., Wang Y. (2023). BART-D2 subtype of EBV encoded BART miRNA cluster 1 region is strongly associated with endemic nasopharyngeal carcinoma. J. Med. Virol..

[83] Chen S.J., Chen G.H., Chen Y.H., Liu C.Y., Chang K.P., Chang Y.S., Chen H.C. (2010). Characterization of Epstein-Barr virus miRNAome in nasopharyngeal carcinoma by deep sequencing. PLoS ONE.

[84] Iizasa H., Wulff B.E., Alla N.R., Maragkakis M., Megraw M., Hatzigeorgiou A., Iwakiri D., Takada K., Wiedmer A., Showe L. (2010). Editing of Epstein-Barr virus-encoded BART6 microRNAs controls their dicer targeting and consequently affects viral latency. J. Biol. Chem..

[85] Lung R.W., Tong J.H., Sung Y.M., Leung P.S., Ng D.C., Chau S.L., Chan A.W., Ng E.K., Lo K.W., To K.F. (2009). Modulation of LMP2A expression by a newly identified Epstein-Barr virus-encoded microRNA miR-BART22. Neoplasia.

